# Novel trigenic *CACNA1C/DES/MYPN* mutations in a family of hypertrophic cardiomyopathy with early repolarization and short QT syndrome

**DOI:** 10.1186/s12967-017-1180-1

**Published:** 2017-04-20

**Authors:** Yanhong Chen, Hector Barajas-Martinez, Dongxiao Zhu, Xihui Wang, Chonghao Chen, Ruijuan Zhuang, Jingjing Shi, Xueming Wu, Yijia Tao, Weidong Jin, Xiaoyan Wang, Dan Hu

**Affiliations:** 1 Department of Cardiology, Wuhan Asia Heart Hospital, Wuhan University, Wuhan, 430022 China; 2 Department of Cardiology, Nantong University, 3rd People’s Hospital of Wuxi Affiliated To Nantong University, 585 Xingyuan Road, Wuxi, 214043 Jiangsu China; 3 Department of Cardiology and Cardiovascular Research Institute, Renmin Hospital of Wuhan University, Wuhan, 430060 China; 4 Masonic Medical Research Laboratory, 2150 Bleecker St, Utica, NY 13501 USA; 5 Molecular Genetics Department, SCRO Chair of Stem Cell Center, Masonic Medical Research Laboratory, 2150 Bleecker St, Utica, NY 13501 USA

**Keywords:** Hypertrophic cardiomyopathy (HCM), Early repolarization (ER), Calcium channels, Genetics, Cardiac resynchronization therapy (CRT)

## Abstract

**Background:**

Hypertrophic cardiomyopathy (HCM) patients with early repolarization (ER) pattern are at higher risk of ventricular arrhythmia, yet the genetic background of this situation has not been well investigated. Here we report novel trigenic mutations detected in a Chinese family of obstructive HCM with ER and short QT syndrome (SQTS).

**Methods:**

Proband and family members underwent detailed medical assessments. DNAs were extracted from peripheral blood leukocytes for genetic screening with next generation method. The functional characterization of the mutation was conducted in TSA201 cells with patch-clamp experiment.

**Results:**

The proband was a 52-year-old male who had a ER pattern ECG in inferioral-lateral leads with atrioventricular block and QTc of 356 ms. He also suffered from severe left ventricular hypertrophy and dysfunction. Targeted sequencing revealed trigenic mutations: c.700G>A/p.E234K in *DES*, c.2966G>A/p.R989H in *MYPN,* and c.5918G>C/p.R1973P in *CACNA1C*. All mutations were also detected in his daughter with ER and mild myocardium hypertrophy. The *CACNA1C*-R1973P mutation caused significant reduction (68.4%) of I_Ca_ compared to *CACNA1C*-WT (n = 14 and 14, P < 0.05). The computer modeling showed that all 3 mutations were highly disease-causing. The proband received the CRT-D (cardiac resynchronizing therapy) implantation, which lowered the left ventricular outflow tract gradient (LVOTG, 124 mmHg pre vs. 27 mmHg post) and restored the LV function (LVEF 40% pre vs. 63% post).

**Conclusions:**

The study reveals a novel *CACNA1C* mutation underlying the unique ER pattern ECGs with SQTS. It also shows the rare trigenic mutations are the pathogenic substrates for the complicated clinical manifestation in HCM patients.

**Electronic supplementary material:**

The online version of this article (doi:10.1186/s12967-017-1180-1) contains supplementary material, which is available to authorized users.

## Background

Hypertrophic cardiomyopathy (HCM) is the most common cause of sudden cardiac death (SCD) in the young as well as in the trained athletes, and is also a critical substrate for heart failure disability at any age. HCM is a clinical heterogeneous but relatively common form of genetic heart defect transmitted as autosomal dominant trait (affecting 1 in 500 people). It is characterized by unexplained cardiac hypertrophy, myocyte disarray, and fibrosis [1]. The yield of genetic testing among HCM cases is as high as ~70%, and comprehensive or targeted (*MYH7, MYBPC3, TNNI3, TNNT2, TPM1*) HCM genetic testing is recommended for patients with HCM and first-degree relatives based on clinical history, family history, and electrocardiographic/echocardiographic phenotype [2, 3]. HCM patients with J point and ST segment elevation are related to higher risk of ventricular arrhythmias and SCD [4]. However, the genetic background of this situation has not been deeply investigated before.

Early repolarization (ER) pattern is defined as J wave (>0.1 mV) with or without ST segment elevation in at least two continuous leads in standard 12-lead ECGs [5]. It has been related with malignant ventricular tachycardia and SCD [6]. ER pattern also has a tendency of heritability [7]. Several ion channel mutations have been linked to ER syndrome, such as genes in I_K,ATP_ and I_Na_ [8–10]. It is worth mentioning that, cardiac calcium channel mutations are related to ER with shortened QTc interval because of its influence on the action potential duration (APD). These patients had higher tendencies for cardiac event and SCD [8].

Here we report a Chinese family of HCM with early repolarization pattern, and borderline short QTc interval. The clinical phenotypes included severe cardiac hypertrophy and left ventricular outflow tract obstruction, left ventricular dysfunction, atrial fibrillation, and sustained ventricular tachycardia. Novel trigenic mutations in *DES*/*MYPN*/*CACNA1C* are detected in the proband and his family. The clinical characteristics and genetic background of the family members are investigated in detail.

## Methods

For details, please see Additional file 1.

### Clinical history

The study was approved by the ethics committee of 3rd People’s Hospital of Wuxi (Wuxi, China) and conducted according to Declaration of Helsinki principles. The informed consents were obtained from all the participants, who belonged to Asian. The clinical assessments included medical history, detailed physical examination, blood tests, electrocardiogram (ECG), ultrasonic cardiogram (UCG), and coronary artery angiography. Patients were clinically diagnosed according to the 2011 ACC/AHA Guideline for the Diagnosis and Treatment of Hypertrophic Cardiomyopathy [2].

### Genetic studies

DNAs were extracted from peripheral blood leukocytes for genetic screenings with next generation method. The identification of known pathogenic variants was based on mutations previously reported to cause cardiovascular disease in the literature. Novel variants considered to be pathogenic were either: (1) stop/frameshift variants; (2) missense mutations positioned in the amino acid conservative region across species; (3) splice-site variations fulfilling the GT-AT rules; or (4) predicted to be possibly damaging or disease-causing by the bioinformatic programs of PolyPhen-2, PROVEAN and MutationTaster2.

The structures of *DES*-E234K and *MYPN*-R989H proteins were modeled with protein structure homology modelling, through the online workspace of SWISS-MODEL (http://swissmodel.expasy.org). The mutated sites were highlighted and visualized using VMD1.9.2 (*University of Illinois at Urbana*-*Champaign*).

### Expanded validation

DNA samples of all the participants were taken for the expanded validations. Coding regions of the mutations identified as described above were amplified by polymerase chain reaction (PCR) for conventional direct sequencing. Purified PCR products were cycle-sequenced on an ABI 3500 Genetic Analyzer (Applied Biosystems, CA). The sequencing results were analyzed by Mutation Surveyor (Softgenetics, PA) and reconfirmed by the same procedure.

### Functional characteristics of calcium mutation

Functional characterization of the *CACNA1C*-R989H mutation was conducted by co-expression of *CACNB2B* and *CACNA2D1* in TSA201 cells, which is a human embryonal kidney with SV40 transformed. Whole cell currents were recorded at room temperature using patch clamp techniques as previously described. Standard whole-cell patch clamp technique was used to measure *CACNA1C* wild type and mutant calcium currents at room temperature (22–24 °C) with the use of an Axopatch 200 B amplifier, Digidata 1440 A and pclamp version 10.4 software (Axon Instruments, Sunnyvale, CA). Microelectrodes were pulled on a P-97 puller (Sutter Instruments, Novato, CA) and fire polished to a final resistance of 1.5–3 MΏ. Series resistance was compensated by 80–85%. Currents were filtered at 1 kHz and digitized at 5 kHz with an eight-pole Bessel filter. Data were analyzed using Clampfit (Axon Instruments, Sunnyvale, CA), Excel (Microsoft, Redmond, WA), and fitted with Origin 8 (OriginLab Corporation, Northampton, MA) software. The steady-state inactivation curve was fitted with a Boltzmann function*: I*_*Ca*_ */I*_*Ca max*_ = *{1* + *exp [(V* − *V*_*1/2*_ *)/k]}* ^−*1*^, where *V*_*1/2*_ and *k* are the half-maximal voltage of inactivation and the slope factor respectively.

### Statistical analysis

All data points are shown as the mean value and bars represent the standard error of the mean. The Student’s unpaired *t* test was performed to determine statistical significance between two groups. *P* < 0.05 was considered to be statistically significant.

## Results

### Clinical history

The proband was a 52-year-old male presented for chest discomfort and stress-induced dyspnea in 2008. The ECG showed sinus bradycardia (46 bpm), I^°^ atrioventricular block (PR interval = 240 ms) and complete left bundle branch block (CLBBB). ER pattern presented in inferior-lateral leads (II, aVF and V4–V6). Convex ST-segment elevations could be seen in lateral leads V4–V6. Other possible causes of abnormal ST-segment elevations were ruled out, including acute myocardial infarction and ventricular aneurysm. The QTc was 356 ms. The UCG showed LV hypertrophy (LVPW 17 mm, IVS 21 mm) with the basal LVOTG of 48 mmHg. The patient was diagnosed with obstructive hypertrophic cardiomyopathy and received drug therapy of beta-blockers (metoprolol tartrate, 47.5 mg qd) hereafter. Because bradycardia and atrioventricular block has gotten worse since then, metoprolol was withdrawn. In 2009, the patient was admitted for aggravated dyspnea and chest discomfort. UCG showed severe LV hypertrophy (LVPW 18 mm, IVS 25 mm) with the LVOTG of 143 mmHg at rest. He received the percutaneous transluminal alcohol septal ablation. The symptoms were relieved and the LVOTG was decreased to 45 mmHg thereafter.

**Fig. 1:**
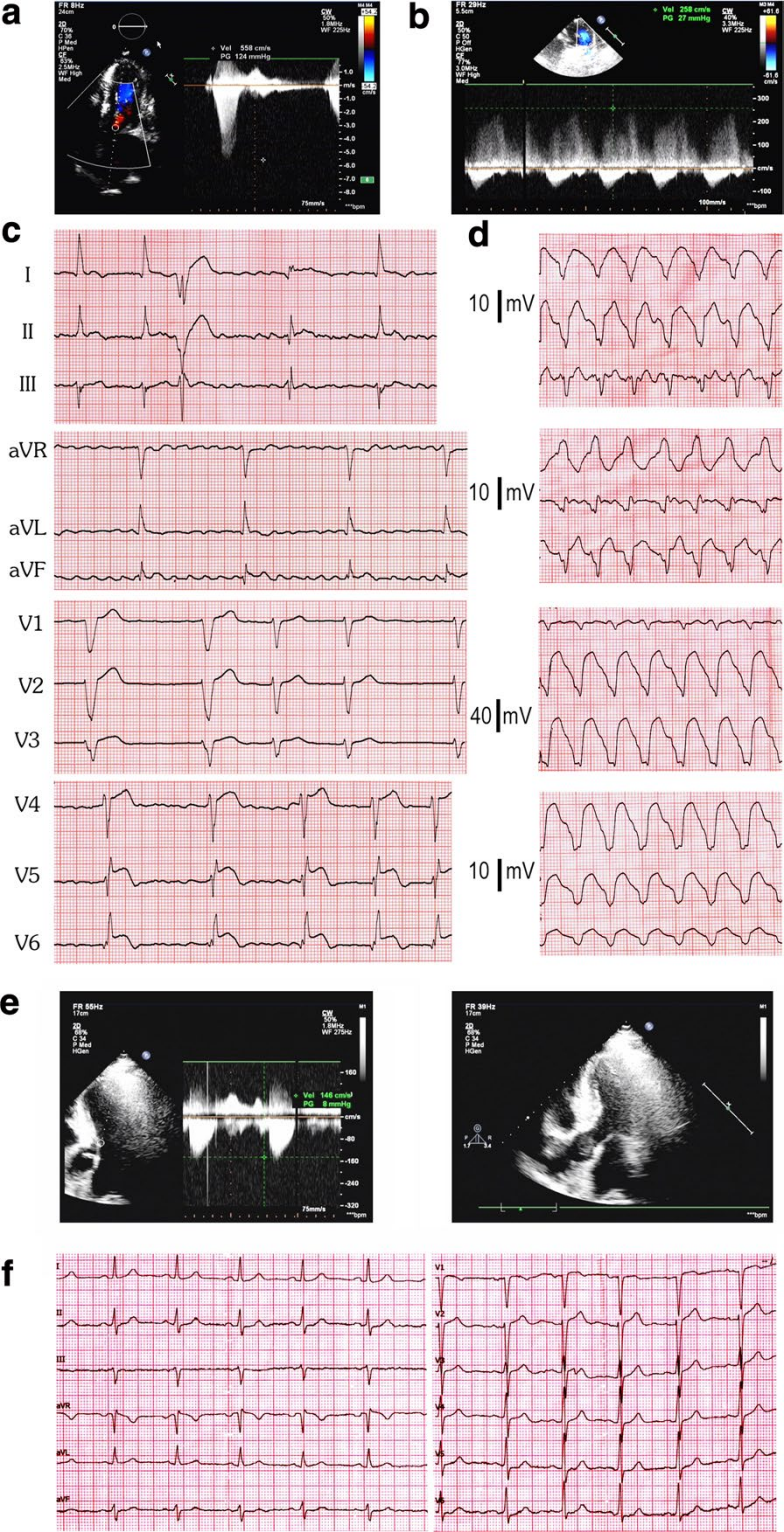
The UCG and ECG of the proband and his daughter. **a** The UCG before the CRT-D implantation. Severe myocardial hypertrophy and left ventricle outflow tract obstruction with the gradient of 124 mmHg. **b** The LVOT obstruction was greatly relieved and the gradient dropped to 27 mmHg after 1 week of the CRT-D implantation. **c** 12 Lead ECG in 2013 showed atrial fibrillation with slow ventricular rates. J waves could be seen in leads I, II, aVL, aVF, and V4–V6, with the amplitudes ranging from 0.2 to 0.4 mV. Covex-shaped ST segment elevation could be seen in lateral leads V4–V6. The QTc averaged 356 ms in V5 and V6 leads. **d** The ECG of the sustained ventricular tachycardia. **e** The UCG of the daughter showed left ventricle hypertrophy and mild LVOT obstructions. **f** The ECG of the daughter showed abnormal Q waves in leads V1–V3, but we didn’t find any ER waves

During the follow-up in 2013, the patient complained of occasional palpitation, dizziness and dyspnea. The ECG showed atrial fibrillation with slow ventricular response of 40–50 bpm. UCG showed the recurrence of LV hypertrophy and increased LVOTG. Transaortic septal myectomy and pacemaker implantation were advised, but the patient refused. In Dec 2013 the patient was presented to the emergency department for continuous palpitation and dyspnea, with compromised blood pressure. The ECG showed persistent ventricular tachycardia (VT) with the heart rate of 150 bpm. The UCG showed LV dysfunction (LVEF, 40%), LV hypertrophy and severe LVOT obstruction (LVOTG, 124 mmHg at rest). After the VT was terminated with intravenous application of lidocaine, he presented slow heart rate (50 bpm) with frequent ventricular premature contractions (PVCs). Prominent J wave presented in leads I, II, aVL, aVF, and V4–V6, with the amplitudes ranging from 0.2 to 0.4 mV. Covex ST-segment elevation could be seen in lateral leads V4–V6. The QTc averaged 356 ms in V5 and V6 leads. Furthermore, VT tended to recur easily. The heart rhythm was stabilized with temporary pacing (lifting the HR to 80 bpm) combined with amiodarone. In January 2014, the patient received the implantation of a cardiac resynchronizing therapy (CRT-D). The symptoms and the heart function were greatly increased thereafter. Interestingly, the LVOTG was dramatically decreased to 27 mmHg. The effectiveness of the CRT-D persisted during the follow-up, and the LVOTG was further lowered to 17 mmHg at 12 months after the implantation. Figure 1a–d showed the ECGs and the UCGs of the index patient.

The patient’s 30-year-old daughter was asymptomatic. The UCG showed left ventricle hypertrophy with LVOTG of 8 mmHg. Her ECG showed abnormal Q waves in leads V1–V3 without ER pattern in any leads (Fig. 1e, f). The patient’s elder sister had no symptoms, and her UCG and ECG were normal. The mother of the proband died suddenly at the age of 70 for unknown origin. Table 1 displayed the UCG parameters of the patient and his family members.

**Table 1:** UCG characteristics of the proband and family members

	Proband							Daughter	Elder sister
	2008	2009	2010	2013	2014 (1 w post CRT-D)	2014 (6 m post CRT-D)	2015 (12 m post CRT-D)		
IVS (mm)	21	25	15	23	23	22	21	13	10
LVPW (mm)	17	18	18	18	23	21	20	13	9
LVOTG (mmHg)	48	143	45	124	27	16	17	8	–
LVEDd (mm)	50	52	50	56	54	54	52	55	55
LVESd (mm)	31	32	31	40	30	31	30	31	34
LVEF %	60	55	60	40	63	60	60	64	60

*IVS* intraventricular septum, *LVPW* left ventricular post wall, *LVOTG* left ventricle outflow tract gradient, *LVEDd* left ventricle end-diastolic diameter, *LVESd* left ventricle end-systolic diameter, *LVEF* left ventricular ejection fraction

### Genetic screening identified candidate mutations

**Fig. 2:**
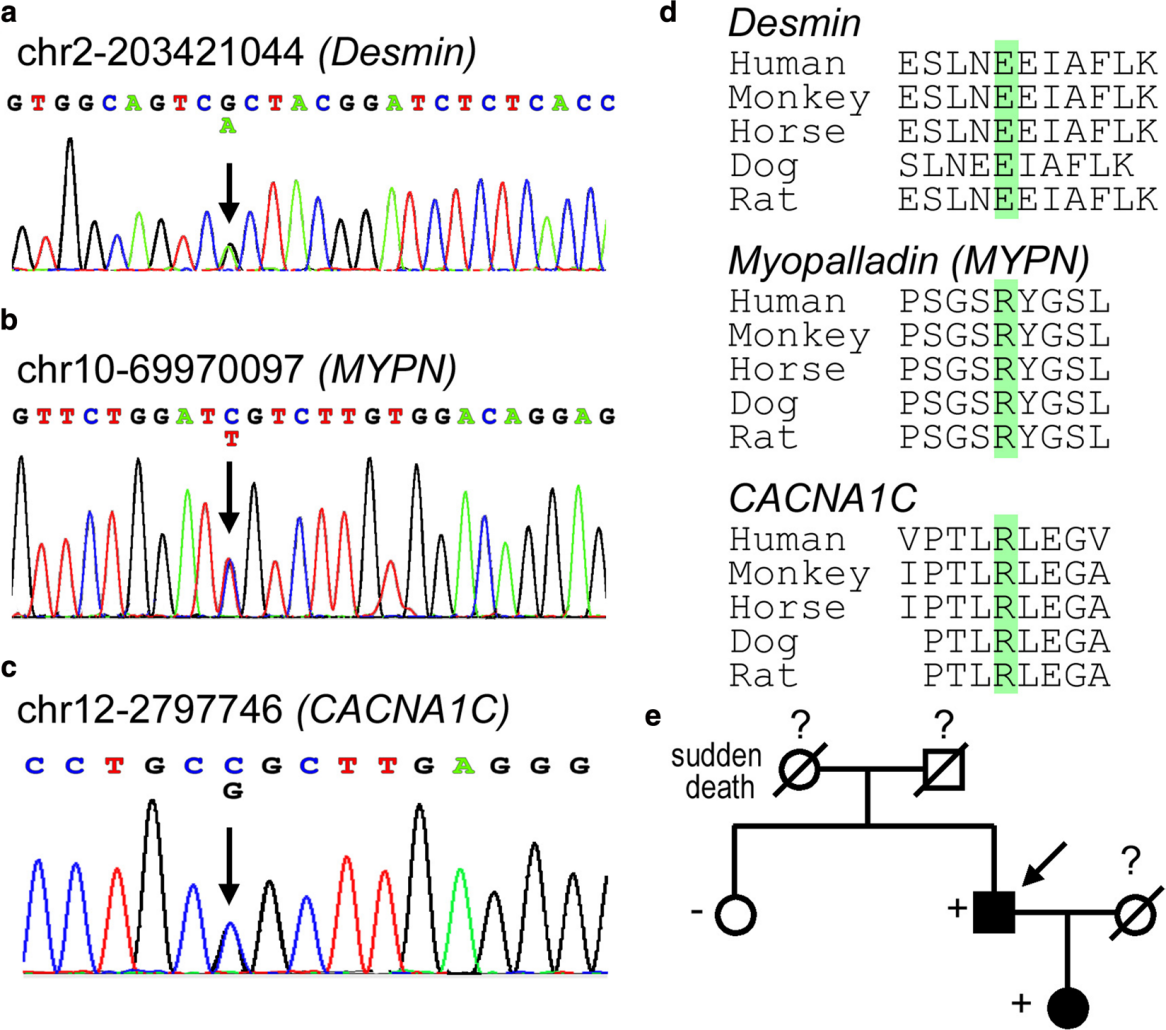
The gene mutations detected in the family members. **a** The mutated site of the desmin (*DES*), c.700G>A/p.E234K. **b** The mutated site of the Myopalladin (*MYPN*), c.2966G>A/p.R989H. **c** The mutated site of the gene *CACNA1C*, c.5918G>C/p.R1973P. **d** The conservation of the mutated site. All 3 sites were highly conserved among different species. **e** The pedigree chart of the family. The symptom-free daughter showed mild myocardial hypertrophy and left ventricle outflow tract obstruction, but her ECG did not show abnormal ST segments elevations. The *DES*, *MYPN* and *CACNA1C* mutations were detected in the daughter but not in the older sister, whose UCG did not show any abnormalities

DNA samples of the proband were applied for targeted exome sequencing of 120 genes implicated in inherited cardiovascular diseases (Additional file 1: Table S1). The average sequencing depths on the targeted regions exceeded 100.0. The sample covered more than 98.0% of the targeted regions. We identified 3 novel missense mutations in the proband: c.700G>A/p.E234K in the exon3 of desmin (*DES*), c.2966G>A/p.R989H in the exon24 of myopalladin (*MYPN*), and c.5918G>C/p.R1973P in exon46 of *CACNA1C* (Fig. 2; Table 2). All mutations were indicated as pathogenic mutation by MutationTaster2, Polyphen2, and PROVEAN. Meanwhile, no pathogenic variant was discovered in typical HCM candidate genes, such as β-myosin heavy chain (*MYH7*), cardiac myosin binding protein-C (*MYBPC3*), α-tropomyosin (*TPM1*), cardiac troponin T (*TNNT2*), myosin regulatory light chain (*MYL2*), myosin essential light chain (*MYL3*), cardiac troponin I (*TNNI3*), and cardiac a-actin (*ACTC1*), Burke et al. [11].

**Table 2:** Genetic mutations carried by the proband

	Position	Sequence	Protein	DNA changes	AA changes	MutationTaster2	Polyphen2	PROVEAN
*CACNA1C*	chr12-2797746	NM_000719	Q13936	c.5918G>C	p.R1973P	Disease causing (prob: 0.999)	Possibly damaging (score: 0.887)	Deleterious (score: −3.153)
*DES*	chr2-220285033	NM_001927	P17661	c.700G>A	p.E234K	Disease causing (prob: 0.999)	Probably damaging (score 0.999)	Deleterious (score: −3.292)
*MYPN*	chr10-69970097	NM_001256268	NP_001243197.1	c.2966G>A	p.R989H	Disease causing (prob: 0.999)	Probably damaging (score 1.000)	Deleterious (score: −2.693)

In the expanded validation test, the elder sister of the proband did not carry any of the mutations detected above. However, the 3 mutations were also detected in his daughter. All the mutated amino acid residues were conserved among different species, indicating the sites were conserved through the evolution.

### Computational analysis of the *DES* and *MYPN* mutations

**Fig. 3:**
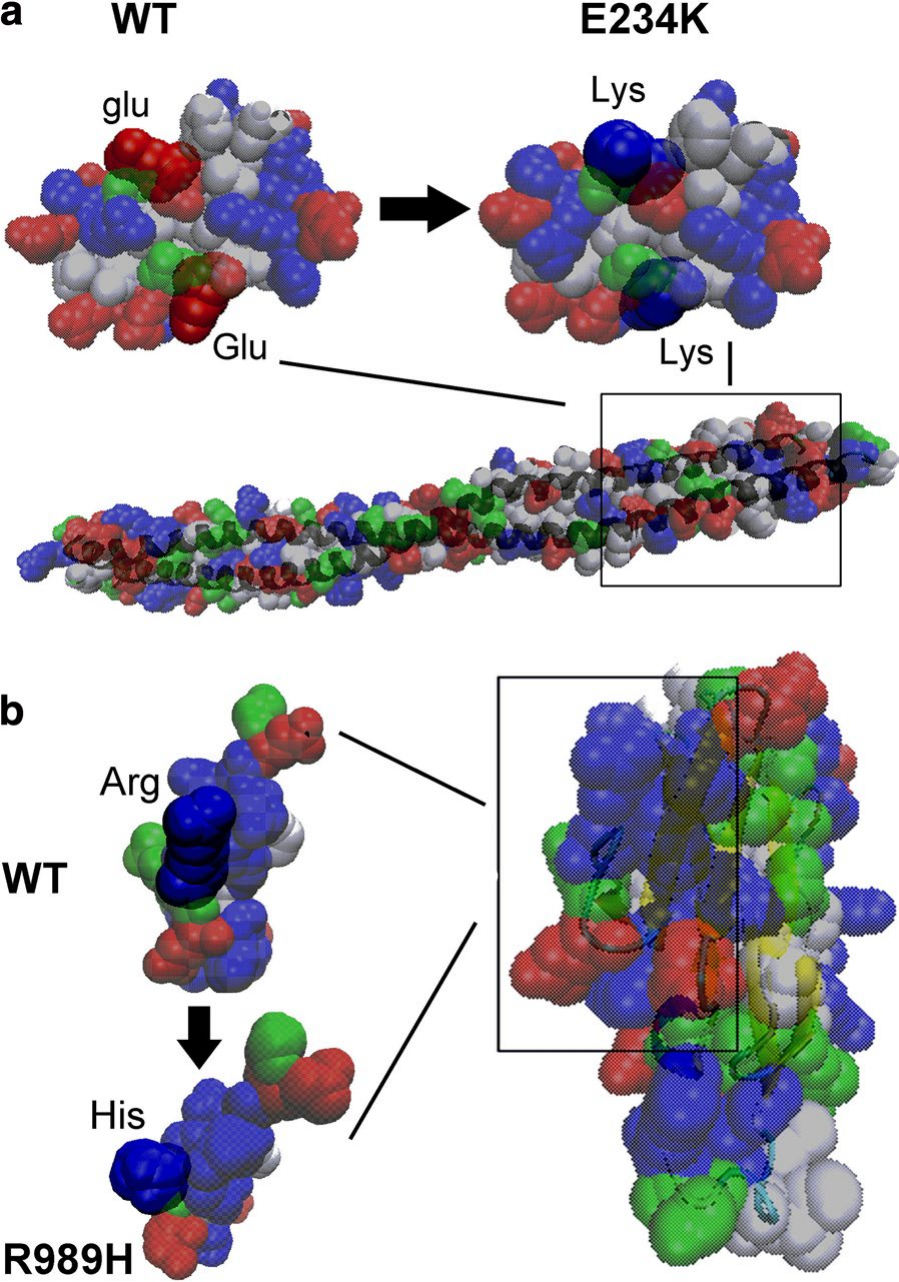
Structure modeling of the mutated domains of Desmin and Myopalladin. **a** The model of *DES* built from the segment of 149th–253th amino-acids sequence. The mutation caused the 234th acidic residue glutamic acid to be replaced by an alkaline residue lysine, which influence the dimerization of protein. **b** The model of *MYPN* built from the segment of 944th–1044th amino-acids sequence. The mutation caused the 989th liphatic amino-acid arginine to be replaced by a heterocyclic residue histidine in mutated myopallin, which changed the protein conformation and was predicted to influence the myopalladin interaction with ACTN

For *DES* and *MYPN,* the structures of the mutated domains were modeled with SWISS-MODEL by protein structure homology modeling. The model of DES was built from the segment of 149-253 amino-acid sequence (Fig. 3a). The c.700G>A mutation in *DES* predicted the 234th acidic glutamic acid to be replaced by an alkaline residue, lysine, which influence the dimerization of the gene. The rod structure of normal Desmin was predicted to be lost. The model of *MYPN* was built from the segment of amino-acid sequence between 944 and 1044 (Fig. 3b). The 989th aliphatic arginine was replaced by a heterocyclic residue, histidine, in mutated myopalladin, which would lead to change the protein conformation and influence the myopalladin interaction with *ACTN*. The function of this region was predicted to be totally lost.

### Functional characterization of R1937P mutant in *CACNA1C*

**Fig. 4:**
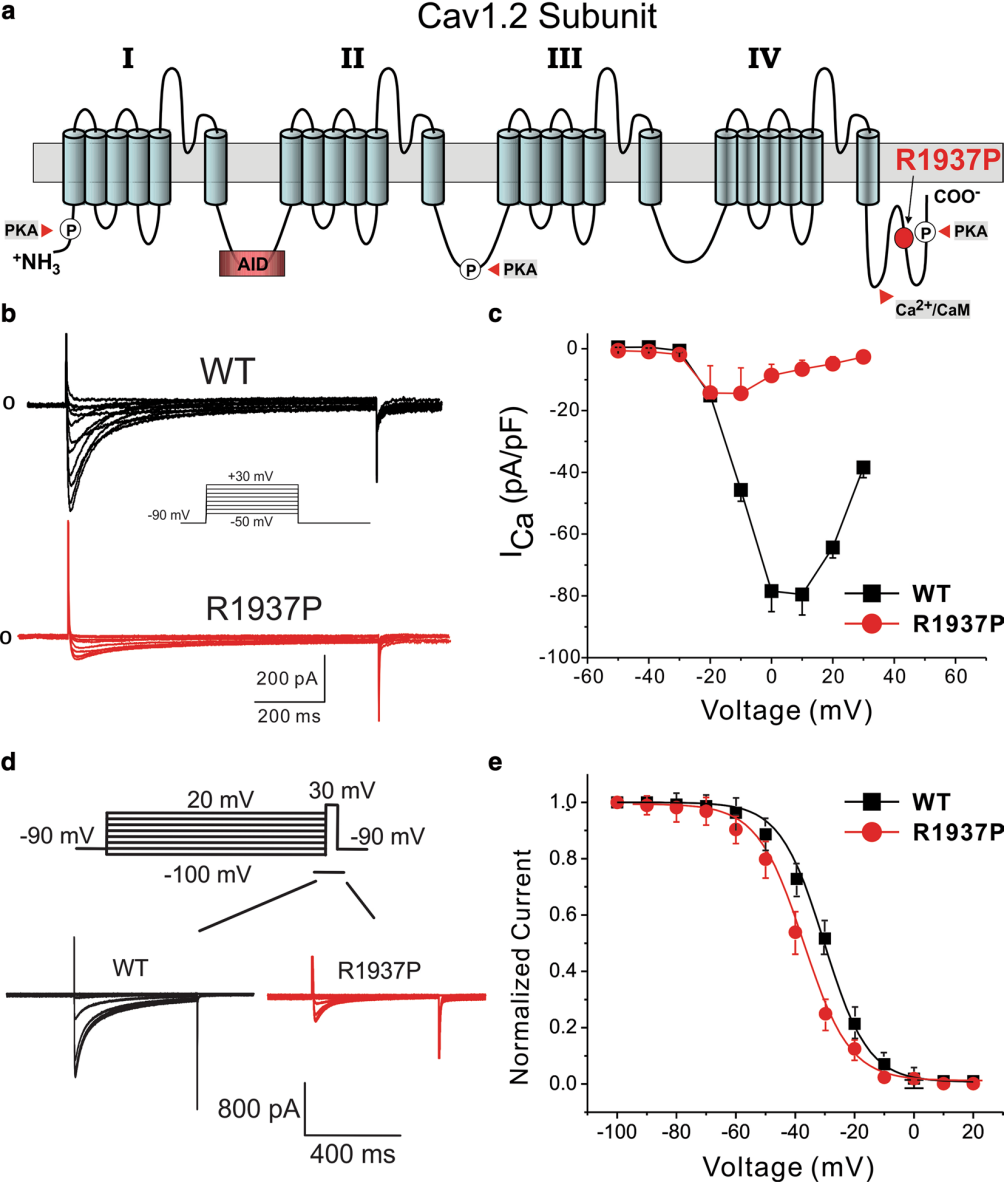
Functional expression studies of the R1973P mutant in *CACNA1C.* **a** 2D Topology of the mutated site. **b** Representative whole-cell calcium current traces (IC_a_) recorded in TSA201 cells transfected with WT-*CACNA1C*and R1973-*CACNA1C* co-expressed with *CACNB2b and CACNA2D1* subunit genes. I_Ca_ traces recorded are in response to the voltage clamp protocol shown at the top inset. **c** Current–voltage relationship (I–V curve) of WT and R1973P variant. Each point data represents the mean ± SEM of 14 experiments. **d** Representative I_Ca_ recorded in response to the voltage clamp protocol shown at the inset on top in TSA201 cells expressing WT or R1973P mutant in *CACNA1C*. Peak currents were normalized to their respective maximum values and plotted against the conditioning potential to obtain the steady-state inactivation curves. **e** R1973P mutant channels showed a significantly more negative mid-inactivation potential compared to WT channels. R1973P (*red circle*) *V*_*1/2*_ = −34.1 ± 0.3 mV, *K* = 6.5 ± 0.3 vs; WT (*black squares*) *V*_*1/2*_ = −30.3 ± 0.3 mV, *K* = 7.4 ± 0.2. Each point data represents the mean ± S.E.M of 4–6 experiments

*CACNA1C* encodes for the α-subunit of cardiac L-type calcium channels. The mutation of c.5918G>C was predicted to cause the 1973th basic arginine to be replaced by the nonpolar/hydrophobic proline in the C-terminal tail of the ion channel (Fig. 4a).

Typical I_Ca_ tracings of voltage-dependent activation from *CACNA1C*-WT and R1937P mutation are shown in Fig. 4b with holding potential at −90 mV to various depolarization potentials (see inset). Analysis of the current–voltage relationship shows that the R1937P mutant dramatically decreased peak current density at −10 mV by 31.6% from −45.7 ± 3.6 pA/pF (WT, n = 14) to −14.45 ± 8.2 pA/pF (R1937P, n = 14, *P* = 0.001), indicating a marked loss-of-function electrophysiological phenotype (Fig. 4c).

Steady-state inactivation was assessed by a standard two-pulse voltage-clamp protocol. Typical tracings of inactivation from *CACNA1C*-WT and -R1937P mutation are shown in Fig. 4d. There was 3.8 negative shift in V_1/2_ of inactivation for R1937P containing channels (−34.1 ± 0.3 mV, n = 4) compared with *CACNA1C*-WT (−30.3 ± 0.3 mV, n = 6, *P* = 0.07, Fig. 4e). The respective *k* slope factor also remained unchanged at 7.4 ± 0.2 (*CACNA1C*-WT, n = 4) and at 6.5 ± 0.3 (*CACNA1C*-R1937P, n = 6; *P* = 0.23).

## Discussion

Hypertrophic cardiomyopathy (HCM) is characterized by asymmetric hypertrophy of interventricular septum, disorganization of cardiomyocytes, as well as myocardial interstitial fibrosis. The basis of HCM has been ascribed to multiple etiologies; however, in 1989 researchers first mapped a genetic mutation for HCM to chromosome [12]. Subsequently, hundreds mutations have been found in HCM patients. Most mutations involve the myofilaments of the cardiac sarcomere (*MYH7*, *MYBPC3*, et al.); however, there is increasing awareness of non sarcomeric mutations as well, such as Z-disk or intracellular calcium modulators.

Comprehensive testing of five HCM genes is strongly recommended to assess patients with HCM by clinical guidelines [2]. Whereas Nextgen sequencing approaches have facilitated much broader testing panels to be widely available, such as the one we employed in this study (Table 2). In both the proband and his daughter, we simultaneously identified trigenic mutations of p.E234K in the *DES*, p.R989H in the *MYPN,* and p.R1973P in *CACNA1C*.

There was few reported trigenic mutation related with cardiomyopathy. One study described a case of HCM with trigenic mutations (LAMA4, PKP2 and TTN) [13], but the ECG characteristics of the patient was not available. On the relationship between gene mutation and QT interval, one study showed that patients with HCM-related gene mutation frequently exhibited QT interval disruption, which was related with increased occurrence of ventricular arrhythmias [14]. Although CACNA1C mutation very likely underlined the shortened QT intervals in our case, it was hard to determine the role of each individual mutation in the genesis of the clinical phenotypes, due to incomplete family history as well as lacking of previous references.

It is well known that QT intervals are slightly prolonged in most HCM patients due to the increased late sodium current and fibrosis [15]. However, the QTc of this proband is significantly short. The intervals from the J points to the peak of T waves were approximately 120 ms. Combined with typical ER in the inferioral-lateral leads, the characteristics of short QT interval might be attributed to the mutation p.R1973P in *CACNA1C*.

*CACNA1C* lie in chromosome 12, coding for α subunit of the L-type calcium channels. The genetic defects in I_Ca,L_ have been linked with LQTS [16], Brugada syndrome [17], ERS [8, 9] and SQTS [18]. Of the reported defects, loss-of-function mutations are extremely rare. In this case, the mutated site was located in the C-terminal of the channel protein (Fig. 4a), which is a “hot region” for loss-of-function mutations. It plays an important role in the Ca^2+^ influx and kinetics procedure, as well as intracellular signal transductions [19, 20]. In order to illuminate the disease-causing ability of the *CACNA1C*-R1973P mutation, heterologous expressions and patch clamping techniques were conducted. We found out that the R1937P mutant dramatically decreased peak current density by ~68%, while exerting no significant influences on the channel kinetics, which is consistent with the previously reported V2014I mutations in the vicinity area [8]. We speculated that the channel current reductions might be due to the trafficking deficiencies of the channel protein. Decreased I_Ca,L_ results in increased net outward currents and shortened cardiomyocyte repolarization period. Due to the transmural discrepancies of outward potassium currents (I_to_, I_K_, etc.), the increase in net outward current result in partial or complete loss of the action potential dome, leading to a transmural voltage gradient that manifests as ER waves. Meanwhile, accelerated repolarization caused by the mutation could lead to subsequent short QT interval in the surface ECG. The SQT4-6 are linked to mutations in different subunits of calcium channel [8, 18]. There is a high prevalence of ER in SQTs [21], suggesting the genetic background of the two diseases may share common fields.

The *CACNA1C* mutation also linked with the slowed AV conduction and bundle branch block, because I_Ca,L_ is the main depolarization current in cardiac conduction cells. Decreased I_Ca,L_ hampers the depolarization of these cells and causes delayed conductions. However, it is also noteworthy that the *DES* mutations could cause atrioventricular blocks [22]. So the conduction delay is probably the outcome of compound genetic defects of both.

The daughter of the patient carries the *CACNA1C* mutation while her ECG presented far less prominent ER, and normal QTc interval. The incomplete penetrance of the *CACNA1C* mutation is probably caused by genetic background or certain factors, such as the gender protection. It is well known that sex hormones have significant influence on the ion channel functions as well as on the cardiac repolarization procedure. Testosterone can shorten cardiomyocyte APDs in guinea pigs, through inhibiting the I_Ca_ and enhancing I_Ks_ [23]. Thus, testosterone’s inhibiting effect on the I_Ca,L_ may worsen the effect of the *CACNA1C* mutation and cause the more severe clinical symptoms of the father. As a matter of fact, ER pattern are more common in male population [24]. Another possible explanation is the matter of age. Experiment on the animals show that the expression of the I_Ca,L_ is decreased with aging [25]. Aging has also been related to decreased expressions of potassium and sodium channels [26, 27]. Collectively, the net inward currents during cardiac repolarization decreased with age. The symptoms of ventricular tachycardia appeared when the patient was in his 50’s, indicating the importance of aging in the pathogenesis. Last but not the least, the degree of the myocardial hypertrophy is also a critical factor. The more severe myocardial hypertrophy of the father may increase the transmural dispersions in the cardiac repolarization and enhance the ST segment elevations, as well as the onset of ventricular tachycardia. Previous studies indicate that variants in sarcomere genes may individually or collectively affect cardiac morphology and function, even without causing overt HCM. HCM is transmitted as a dominant trait. However, penetrance is incomplete and lowest at young ages [11]. Identical HCM mutations in the same pedigree can produce distinct LVH morphologies, varying amounts of myocardial fibrosis, and differing susceptibility to arrhythmias. Genetic modifiers, epigenetic differences, and unique environmental factors are likely to influence these variables. But without long term follow-up and deeper translational research, especially more information form the pedigree, it is hard for us to deduce which assumption is the major cause of ventricular arrhythmia. The proband suffered from severe obstructive HCM and his daughter also showed mild myocardial hypertrophy. The *CACNA1C* mutations have been related to HCM in previous reports. However, there were two other mutations detected in this family, which might be part of the pathogenesis of the HCM. The *DES* gene is located in chromosome 2, coding for desmin protein. Desmin is the type III intermediate filament protein with the molecular weight of 53.5 kD of 470 residues, which is expressed in various types of muscle cells. In the heart, it integrates the Z disc, the sarcolemma and the myocyte nucleus. Mutations in the *DES* gene are the causes of the desmin-related myopathies (DRM) [28]. Recent reports linked *DES* mutations with arrhymogenic right ventricular cardiomyopathy (ARVC) [29] and dilated cardiomyopathy (DCM) [30]. *DES* mutations are rarely associated with HCM except in few sporadic cases [31]. The *MYPN* gene is a 145.2 kDa protein of 1320 residue for encoding myopalladin, which is located in chromosome 10. Myopalladin is the component of the myofilament, connecting the Z disc, the sarcolemma and the myocyte nucleus. The myopalladin participate in the regulations of the contraction and adhension of the cardiomyocytes, as well as the gene expressions. Also, the mutations in *MYPN* were linked with DCM, HCM and restrictive cardiomyopathy (RCM) [32]. Both desmin and myopalladin were important components of the cytoskeleton. The dual mutations cause instability of the sarcomeres and decreased contraction efficiencies. On the other hand, the Ca^2+^ cycling exert significant impact on the sarcomere contractions. Intracellular Ca^2+^ level is crucial for the normal contraction of cardiomyocytes [33]. The impaired function of I_Ca,L_ lead to decreased influx of Ca^2+^ ions, influencing the intracellular Ca^2+^ cycling and reduce the efficiency of sarcomere contraction. The impaired cytoskeletons caused by digenic *DES/MYPN* mutations may also hamper the *CACNA1C* expressions. Thus, the three mutations work together in the impairment of the sarcomere functions and subsequent onset of cardiomyopathy.

The managements of HCM include optimized drug therapy and septal reduction therapy, when the LVOT gradients exceed 50 mmHg with clinical symptoms like dyspnea or chest pain. Either surgical myectomy or septal ablation is generally effective in reducing LOVT obstructions and relieving clinical symptoms. The patient developed refractory LV hypertrophy and severe LVOT obstruction 3 years after the septal ablation, due to those mutations. Interestingly, the CRT significantly reduces the outflow tract gradient. The CRT was not considered as the standard therapy for HCM patients [2], but there are some reports on the effectiveness of resynchronization therapy on reducing the outflow tract gradients in HCM patients [34–36]. Although the mechanism is not clear yet, some proposes that the biventricular pacing changed the sequence of the ventricular excitation, which helps to reduce the outflow tract gradient and to reverse the remodeling of the left ventricles [35]. Our report adds clinical support to the effectiveness of the CRT on reducing the outflow tract gradients, which might be a promising strategy for the treatment of HCM patients.

The daughter gave birth to a boy recently. Unfortunately, she refused genetic screening for the baby boy. To observing the progressive nature of the disease, we plan to follow the daughter as well as her son continuously. Meanwhile, we plan to build human induced pluripotent stem cells (hiPSC) from skin tissue of the proband and family members, to replicating the situation and perform further research on stem cell level with all the genetic features in this pedigree.

In this study, we reported a family with ER, SQTSs and HCM. Novel *CACNA1C* mutation is the pathogenic substrate of the electrophysiological as well as structural abnormities. Meanwhile the rare trigenic mutations make the clinical manifestation complicated and aggravated in this HCM family. At last, the study also suggests the effectiveness of CRT-D on reducing the LVOTG of septal ablation refractory hypertrophic cardiomyopathy.

## Additional file

**Additional file 1: Table S1.** List of genes sequenced for the proband.

## Abbreviations

*CACNA1C*: α1C subunit of calcium voltage-gated channel
CLBBB: complete left bundle branch block
*DES*: desmin
ECG: electrocardiogram
ER: early repolarization
HCM: hypertrophic cardiomyopathy
hiPSC: human induced pluripotent stem cells
*MYPN*: myopalladin
SCD: sudden cardiac death
SQTS: short QT syndrome
UCG: ultrasonic cardiogram
