# Erratum to: Novel trigenic *CACNA1C/DES/MYPN* mutations in a family of hypertrophic cardiomyopathy with early repolarization and short QT syndrome

**DOI:** 10.1186/s12967-017-1203-y

**Published:** 2017-05-11

**Authors:** Yanhong Chen, Hector Barajas‑Martinez, Dongxiao Zhu, Xihui Wang, Chonghao Chen, Ruijuan Zhuang, Jingjing Shi, Xueming Wu, Yijia Tao, Weidong Jin, Xiaoyan Wang, Dan Hu

**Affiliations:** 1 Department of Cardiology, Wuhan Asia Heart Hospital, Wuhan University, Wuhan, 430022 China; 2 Department of Cardiology, Nantong University, 3rd People’s Hospital of Wuxi Affiliated to Nantong University, 585 Xingyuan Road, Wuxi, 214043 Jiangsu China; 3 Department of Cardiology and Cardiovascular Research Institute, Renmin Hospital of Wuhan University, Wuhan, 430060 China; 4 Masonic Medical Research Laboratory, 2150 Bleecker St, Utica, NY 13501 USA; 5 Molecular Genetics Department, SCRO Chair of Stem Cell Center, Masonic Medical Research Laboratory, 2150 Bleecker St, Utica, NY 13501 USA

## Erratum to: J Transl Med (2017) 15:78 DOI 10.1186/s12967-017-1180-1

In the original publication of this article [1], Figure 2 is incorrect. In this Erratum the original figure (Fig. 1) and the correct figure (Fig. 2) are published.

**Fig. 1:**
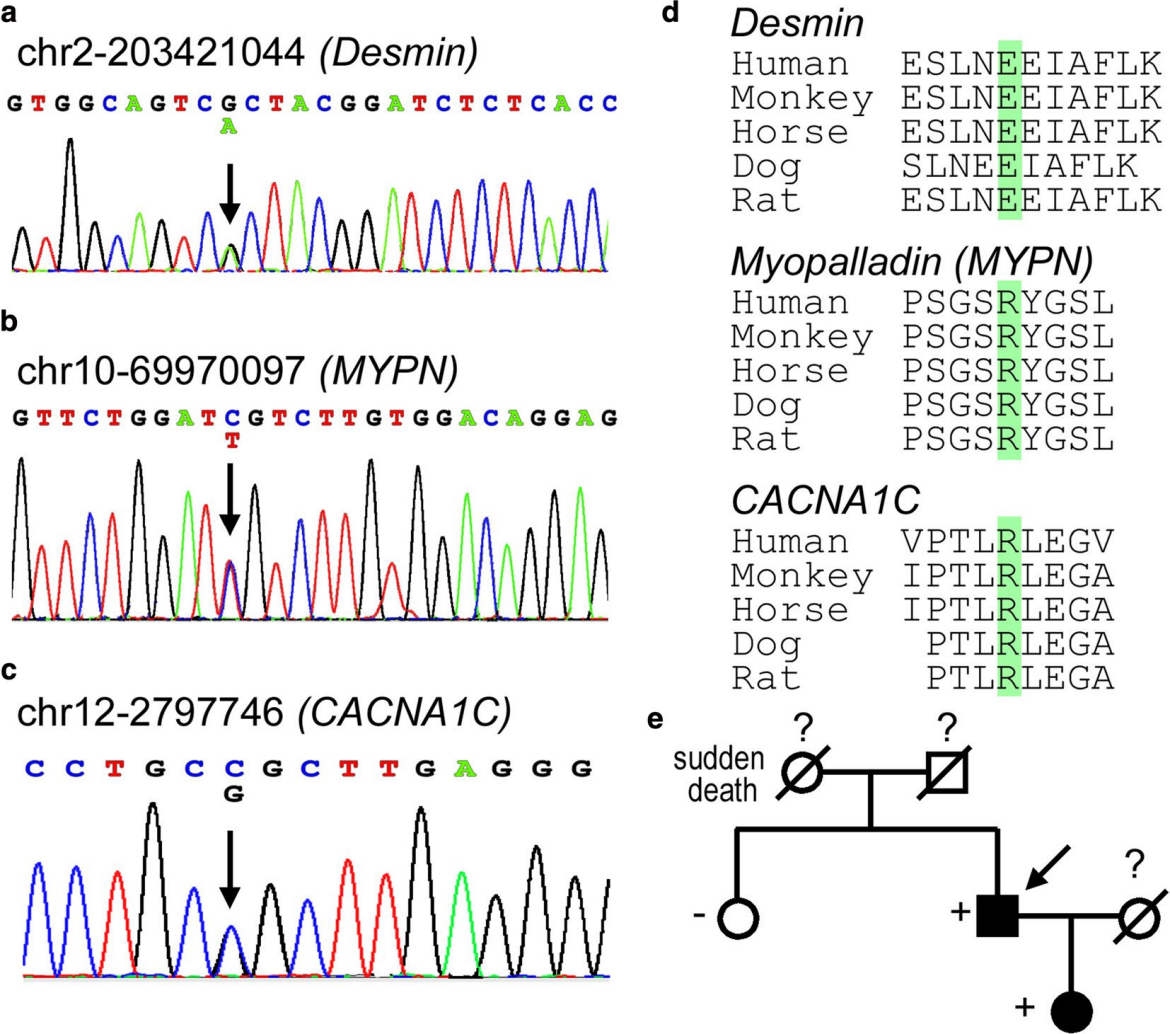
Original version of Fig. 2 as published on 20 April 2017

**Fig. 2:**
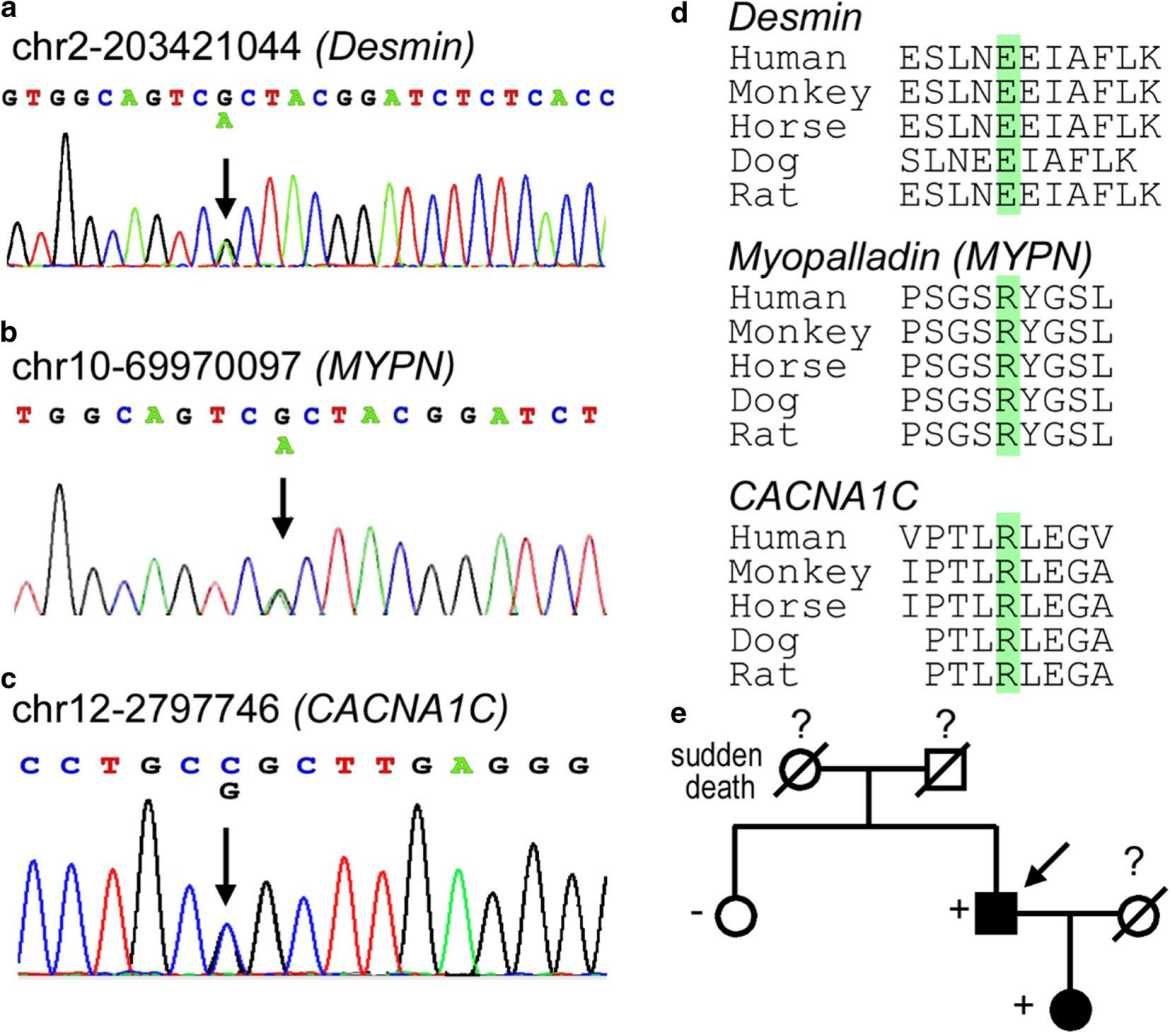
Corrected version of Figure 2; panel **b** has been updated
