# Supplementary material for: Novel trigenic CACNA1C/DES/MYPN mutations in a family of hypertrophic cardiomyopathy with early repolarization and short QT syndrome
Source: J Transl Med. 2017 Apr 20;15:78. doi: 10.1186/s12967-017-1180-1 (PMC5399316; doi:10.1186/s12967-017-1180-1)
Supplement: Supplementary file 1 — Additional file 1: Table S1. List of genes sequenced for the proband. [file 12967_2017_1180_MOESM1_ESM.doc]

# JTRM-D-17-00060

# Novel Trigenic *CACNA1C/DES/MYPN* Mutations in a Family of Hypertrophic Cardiomyopathy with Early Repolarization and Short QT Syndrome

*Short Title – Chen, et al: Novel Trigenic Mutations in HCM family ER and SQTS*

#

# Online Supplement

**Online Methods**

**Clinical history**

The study was approved by the ethics committee of 3rd People’s Hospital of Wuxi (Wuxi, China) and conducted according to Declaration of Helsinki principles. The informed consents were obtained from all the participants, who belonged to Asian. The clinical assessments included history collection, detailed physical examination, blood tests, electrocardiogram (ECG), ultrasonic cardiogram (UCG), and coronary artery computer tomography angiography. Patients were clinically diagnosed according to the 2011 ACC/AHA Guideline for the Diagnosis and Treatment of Hypertrophic Cardiomyopathy.

**Genetic studies**

**DNA extraction, Target region capture, and Next Generation Sequencing**

Genomic DNA was extracted from peripheral blood lymphocytes by standard procedures using QIAamp DNA Bloodmini kits (Qiagen, Germany). One µg genomic DNA was fragmented by Covarissonicator (Covaris S2, USA) to sizes of 150-300 bp and then purified. The blunt ends of the purified DNA fragments were repaired, and A-tailing was added. The fragments were ligated overnight using standard Illumina paired- end (PE) adapter. The ligated products were then amplified through 4-cycle polymerase chain reactions (PCRs) using PE primers containing 8 bp index tags. The purified PCR products containing 3 µg DNA were hybridized to the GenCapTM probe (in solution) at 65°C for 22 hours using a PCR machine. The products were bound to a rotator for 1 hour at room temperature using DynalMyone Streptavidin C1 magnetic beads (Invitrogen, USA), which had been activated beforehand, and the products were then washed with buffer according to the kit manual. The captured DNA libraries were amplified using 15-cycle PCRs, purified, and subsequently eluted in a 30 µl volume and subjected to Agilent 2100 Bioanalyzer and quantitative PCR to estimate the magnitude of enrichment. The final captured DNA libraries were sequenced using the Illumina HiSeq2000 Analyzers as PE 90 bp reads (following the manufacturer’s standard cluster generation and sequencing protocols), providing an average coverage depth for each sample of at least 100-fold.

**Data filtering and analysis**

Image analysis, error estimation, and base calling were performed using the Illumina pipeline (version 1.3.4) with default parameters. Indexed primers were used to identify the different samples in the primary data. All unqualified reads (defined as reads either polluted by adapter, containing more than 10% nucleotides out of read length, having an average quality of less than 10, or having 50% bases with a quality value less than 5) were removed using a local dynamic programming algorithm. The remaining reads were aligned to the reference human genome (UCSC hg19) using Burrows-Wheeler Alignment Tool (BWA-0.5.9). Next, SNPs and indels were identified using SOAPsnp software 2.0 and SAMtools v1.4 while using the recommended parameters.

**Functional annotation of genetic variants**

The variants were functionally annotated using an in-house pipeline as well as the reported frequencies available from public databases (dbSNP 135, HapMap database, 1000 genome variants database, and a local control database) and categorized into either missense, nonsense, splice-site, insertion, deletion, synonymous or noncoding mutations. For all variants, the results were filtered using a quality value of single base sequencing ≥ 20. The variants were filtered to potential mutation candidates through: 1). Functional variants (insertion/deletion: in CDS and splicing region, SNP: nonsense, splice site and missense) and 2). Variants with an allele frequency below 0.01 in either of the public databases mentioned above.

The identification of known pathogenic variants was based on mutations previously reported to cause cardiovascular disease in the literature. Novel variants considered to be pathogenic were either: 1) stop/frameshift variants; 2) missense mutations positioned in the amino acid conservative region across species; 3) splice-site variations fulfilling the GT-AT rules; or 4) predicted to be possibly damaging or disease-causing by the bioinformatic programsPolyPhen-2 (<http://genetics.bwh.harvard.edu/pph2/>), PROVEAN and MutationTaster (http://www.mutationtaster.org/;) .

The structures of E234K-*DES* and R989H-*MYPN* were modeled with protein structure homology modelling, through the online workspace of SWISS-MODEL (*http://swissmodel.expasy.org*) . The mutated sites were marked and visualized using VMD1.9.2 (*University of Illinois at Urbana-Champaign*).

**Expanded Validation**

DNA samples of all the participants were taken for the expanded validations. Coding regions of the mutations identified as described above were amplified by polymerase chain reaction (PCR) for conventional direct sequencing. Purified PCR products were cycle-sequenced on an ABI 3500 Genetic Analyzer (AppliedBiosystems, CA). The sequencing results were analyzed by Mutation Surveyor (Softgenetics, PA) and reconfirmed by the same procedure.

**Cell Biology and Functional Studies**

Functional characterization of the R1937P-*CACNA1C* mutation was conducted by co-expression of *CACNB2B* and *CACNA2D1* in TSA201 cells. Whole cell currents were recorded at room temperature using patch clamp techniques as previously described. Standard whole-cell patch clamp technique was used to measure *CACNA1C* wild type and mutant calcium currents at room temperature (22-24^o^C) with the use of an Axopatch 200B amplifier, Digidata 1440A and pclamp version 10.4 software (Axon Instruments, Sunnyvale, CA). The extracellular (bath) solution contained (mmol/L): 130 NMDG, 5 KCl, 15 CaCl_2_, 1 MgCl_2_, 5 mM TEA-Cland 10 HEPES, pH adjusted to 7.35 with HCl. The pipette solution contained (mmol/L): 120 CsCl, 2 MgCl_2_, 10 EGTA, 2 MgATP, 5 CaCl_2_ and 10 HEPES, pH adjusted to 7.25 with CsOH. Microelectrodes were pulled on a P-97 puller (Sutter Instruments, Novato, CA) and fire polished to a final resistance of 2-3 MΏ. Series resistance was compensated by 80-85%. Currents were filtered at 1 kHz and digitized at 5 kHz with an eight-pole Bessel filter. The voltage dependence of activation and inactivation, were determined using voltage-clamp protocols described in the relevant figures. Data were analyzed using Clampfit (Axon Instruments, Sunnyvale, CA), Excel (Microsoft, Redmond, WA), and fitted with Origin 8 (OriginLab Corporation, Northampton, MA) software. The steady-state inactivation curve was fitted with a Boltzmann function: I_Ca_/I_Camax_={1+exp [(V-V_1/2_)/k]}^-1^, where V_1/2_ andk are the half-maximal voltage of inactivation and the slope factor respectively.

**Statistical Analysis**

All data points are shown as the mean value and bars represent the standard error of the mean. The Student’s t-test was performed to determine statistical significance between two groups. *P*<0.05 was considered to be statistically significant.

**Online Table 1. List of genes sequenced for the proband**

| **gene** | **chromosome** | **gene** | **chromosome** | **gene** | **chromosome** |
| --- | --- | --- | --- | --- | --- |
| *ABCC9* | 12p12.1 | *GDF1* | 19p12 | *NPPA* | 1p36.2 |
| *ACTA2* | 10q22-q24 | *GJA1* | 6q21-q23.2 | *PKP2* | 12p11 |
| ***ACTC1*** | **15q14** | ***GLA*** | Xq22 | ***PLN*** | **6q22.1** |
| ***ACTN2*** | **1q42-q43** | *GLMN* | 1p22-p21 | ***PRKAG2*** | **7q36** |
| *ACVRL1* | 12q11-q14 | *GNAI2* | 3p21 | *PRKAR1A* | 17q23-q24 |
| *AKAP10* | 17p11.1 | *GPD1L* | 3p22.3 | *RASA1* | 5q13.3 |
| *AKAP9* | 7q21-q22 | *HCN4* | 15q24-q25 | *RBM20* | 10q25.2 |
| *ANK2* | 4q25-q27 | *HTRA1* | 10q25.3-q26.2 | *RPS6KA3* | Xp22.2-p22.1 |
| ***ANKRD1*** | **10q23.31** | *JAG1* | 20p12 | *RYR2* | 1q42.1-q43 |
| *APP* | 21q21 | *JAK2* | 9p24 | *RYR2* | 1q42.1-q43 |
| *BAG3* | 10q25.2-q26.2 | *JUP* | 17q21 | *SCN1B* | 19q13.1 |
| *BLM* | 15q26.1 | *KCNA5* | 12p13 | *SCN2B* | 11q23.3 |
| *BMPR2* | 2q33 | *KCNE2* | 21q22.1 | *SCN3B* | 11q24.1 |
| *CACNA1C* | 12p13.3 | *KCNE3* | 11q13-q14 | *SCN4B* | 11q23.3 |
| *CACNB2* | 10p12 | *KCNH2* | 7q35-q36 | *SCN5A* | 3p22.2 |
| *CASQ2* | 1p13.3-p11 | *KCNJ2* | 17q23.1-q24.2 | *SCO2* | 22q13 |
| ***CAV3*** | **3p25** | *KCNMB1* | 5q34 | *SGCD* | 5q33 |
| *CFC1* | 2q21.1 | *KCNQ1* | 11p15.5 | *SNTA1* | 20q11.2 |
| *COL4A1* | 13q34 | ***LAMP2*** | Xq24 | ***TAZ*** | **Xq28** |
| ***COX15*** | **10q24** | *LDB3* | 10q22.2-q23.3 | *TBX1* | 22q11.2 |
| *CRELD1* | 3p25.3 | *LMNA* | 1q21.2 | *TBX20* | 7p15-p14 |
| ***CRYAB*** | **11q23.1** | *LRP6* | 12p13.3-p11.2 | *TBX5* | 12q24.1 |
| *CRELD1* | 3p25.3 | *LRP8* | 1p34 | ***TCAP*** | **17q12** |
| ***CSRP3*** | **11p15.1** | *MED13L* | 12q24 | *TFAP2B* | 6p12 |
| *CTF1* | 16p11.2 | *MEF2A* | 15q26 | *TGFB3* | 14q24 |
| *DES* | 2q35 | ***MYBPC3*** | **11p11.2** | *TGFBR1* | 9q22 |
| *DMD* | Xp21.2 | *MYCN* | 2p24.1 | *TGFBR2* | 3p22 |
| *DPP6* | 7q36.2 | *MYH11* | 16p13.13-p13.12 | *TLL1* | 4q32-q33 |
| *DSC2* | 18q12.1 | ***MYH6*** | **14q12** | *TMEM43* | 3p25 |
| *DSG2* | 18q12.1-q12.2 | ***MYH7*** | **14q12** | ***TNNC1*** | **3p21.3-p14.3** |
| *DSP* | 6p24 | ***MYL2*** | **12q23-q24.3** | *TNNI3* | 19q13.4 |
| *DTNA* | 18q12.1-q12.2 | ***MYL3*** | **3p** | ***TNNT2*** | **1q32** |
| *ELN* | 7q11.2 | ***MYLK2*** | 20q13.31 | ***TPM1*** | **15q22.1** |
| *ENG* | 9q34.1 | ***MYO6*** | 6q13 | *TRPM4* | 19q13.2-q13.3 |
| *ENPP1* | 6q22-q23 | ***MYOZ2*** | 4q26-q27 | *TSPYL1* | 6q22-q23 |
| *EYA4* | 6q23 | *MYPN* | 10q21.3 | ***TTN*** | **2q31** |
| *F12* | 5q33-qter | ***NEXN*** | **1p32-p31** | ***TTR*** | 18q12.1 |
| *F5* | 1q23 | *NKX2-5* | 5q34 | ***VCL*** | **10q22.1-q23** |
| *FKTN* | 9q31 | *NKX2-6* | 8p21 | *VKORC1* | 16p11.2 |
| *GATA4* | 8p23.1-p22 | *NOTCH1* | 9q34.3 | *WT1* | 11p13 |

Note: bold letters indicate known HCM susceptible genes.

**References**
